# Supplementary material for: From sequence to enzyme mechanism using multi-label machine learning
Source: BMC Bioinformatics. 2014 May 19;15:150. doi: 10.1186/1471-2105-15-150 (PMC4229970; doi:10.1186/1471-2105-15-150)
Supplement: Additional file 2 — Java code of ml2db. Additional file ml2db_code.tar.gz contains the Java source code to run the multi-label machine learning experiments and save the results to database. The code’s Javadoc is included. [file 1471-2105-15-150-S2.zip › additional file 2/ml2db/ml2db/doc/mulan/classifier/neural/MMPUpdateRuleBase.html]

MMPUpdateRuleBase


JavaScript is disabled on your browser.


- Overview
- Package
- Class
- Use
- Tree
- Deprecated
- Index
- Help

- Prev Class
- Next Class

- Frames
- No Frames

- All Classes

- Summary:
- Nested |
- Field |
- Constr |
- Method

- Detail:
- Field |
- Constr |
- Method


mulan.classifier.neural

## Class MMPUpdateRuleBase

- java.lang.Object
- - mulan.classifier.neural.MMPUpdateRuleBase

- All Implemented Interfaces:
  :   ModelUpdateRule

  Direct Known Subclasses:
  :   MMPMaxUpdateRule, MMPRandomizedUpdateRule, MMPUniformUpdateRule

  ---

    

  ```
  public abstract class MMPUpdateRuleBase
  extends java.lang.Object
  implements ModelUpdateRule
  ```

  The base class of update rules for `MMPLearner`. The base class implements the
  `ModelUpdateRule` interface and provides a common logic shared among update rules
  for `MMPLearner`. More information on uprate rules logic can be found in paper referenced
  by `MMPLearner`.

  Version:
  :   2012.02.27

  Author:
  :   Jozef Vilcek

  See Also:
  :   `MMPLearner`

- - ### Constructor Summary

    Constructors

    Constructor and Description

    `MMPUpdateRuleBase(java.util.List<Neuron> perceptrons,
    RankingLossFunction loss)`

    Creates a new instance of `MMPUpdateRuleBase`.
  - ### Method Summary

    Methods

    | Modifier and Type | Method and Description |
    | --- | --- |
    | `double` | `process(DataPair example, java.util.Map<java.lang.String,java.lang.Object> params)` Process the training example and performs a model update when suitable. |

    - ### Methods inherited from class java.lang.Object

      `equals, getClass, hashCode, notify, notifyAll, toString, wait, wait, wait`

- - ### Constructor Detail


    - #### MMPUpdateRuleBase

      ```
      public MMPUpdateRuleBase(java.util.List<Neuron> perceptrons,
                       RankingLossFunction loss)
      ```

      Creates a new instance of `MMPUpdateRuleBase`.

      Parameters:
      :   `perceptrons` - the list of perceptrons, representing the model, which will receive updates.
      :   `loss` - the lossFunction measure used to decide when the model should be updated by the rule
  - ### Method Detail


    - #### process

      ```
      public final double process(DataPair example,
                   java.util.Map<java.lang.String,java.lang.Object> params)
      ```

      **Description copied from interface: `ModelUpdateRule`**

      Process the training example and performs a model update when suitable.
      The decision when to perform model update is carried by the update rule
      (e.g. when the model response is not within an acceptable boundaries from
      the true output for given example).

      **Specified by:**
      :   `process` in interface `ModelUpdateRule`

      Parameters:
      :   `example` - the input example
      :   `params` - the additional parameters for an update.

      Returns:
      :   the error measure of the model response for given input pattern
          and specified true output.


- Overview
- Package
- Class
- Use
- Tree
- Deprecated
- Index
- Help

- Prev Class
- Next Class

- Frames
- No Frames

- All Classes

- Summary:
- Nested |
- Field |
- Constr |
- Method

- Detail:
- Field |
- Constr |
- Method
